# Low temperature modifies seedling leaf anatomy and gene expression in *Hypericum perforatum*

**DOI:** 10.3389/fpls.2022.1020857

**Published:** 2022-09-27

**Authors:** Hongyan Su, Ling Jin, Mengfei Li, Paul W. Paré

**Affiliations:** ^1^ State Key Laboratory of Arid Land Crop Science, Gansu Agricultural University, Lanzhou, China; ^2^ College of Pharmacy, Gansu University of Chinese Medicine, Lanzhou, China; ^3^ Department of Chemistry and Biochemistry, Texas Tech University, Lubbuck, TX, United States

**Keywords:** *hypericum perforatum*, temperature, green tissue, dark gland, secretory cell, hypericin

## Abstract

*Hypericum perforatum*, commonly known as St John’s wort, is a perennial herb that produces the anti-depression compounds hypericin (Hyp) and hyperforin. While cool temperatures increase plant growth, Hyp accumulation as well as changes transcript profiles, alterations in leaf structure and genes expression specifically related to Hyp biosynthesis are still unresolved. Here, leaf micro- and ultra-structure is examined, and candidate genes encoding for photosynthesis, energy metabolism and Hyp biosynthesis are reported based on transcriptomic data collected from *H. perforatum* seedlings grown at 15 and 22°C. Plants grown at a cooler temperature exhibited changes in macro- and micro-leaf anatomy including thicker leaves, an increased number of secretory cell, chloroplasts, mitochondria, starch grains, thylakoid grana, osmiophilic granules and hemispherical droplets. Moreover, genes encoding for photosynthesis (64-genes) and energy (35-genes) as well as Hyp biosynthesis (29-genes) were differentially regulated with an altered growing temperature. The anatomical changes and genes expression are consistent with the plant’s ability to accumulate enhanced Hyp levels at low temperatures.

## 1 Introduction

*Hypericum perforatum* L. (St John’s wort) is a perennial herb widely distributed in Europe, Asia, Northern Africa and Northern America (Bagdonaitė et al., 2010). Aerial parts contain the metabolites hypericin (Hyp) and hyperforin that are used in traditional medicine as anti-depression, anti-viral, anti-microbial and anti-tumor agents, as well as other plant constituents such as flavonoids, tannins and volatile oils (Barnes et al., 2001; Napoli et al., 2018).

St John’s wort has traditionally been used as an external anti-infammatory and healing remedy for the treatment of swellings, wounds and burns. It is of interest recently due to new and important therapeutic applications (Bombardelli and Morazzoni, 1995; Nahrstedt and Butterweck, 1997; Erdelmeier, 1998). The species is characterized by the presence of different types of secretory structure: translucent glands or cavities, black nodules and secretory canals (Ciccarelli et al., 2001). The frequency and diversity of these secretory structures is evidence of the intense secretory activity of the species. Previous studies have found that *H. perforatum* growth and Hyp accumulation are affected by the germplasm source (Couceiro et al., 2006; Soták et al., 2016; Zhang et al., 2021) as well as environmental factors such as light quality (Germ et al., 2010; Najafabadi et al., 2019; Tavakoli et al., 2020; Karimi et al., 2022), drought (Gray et al., 2003), and temperature (Zobayed et al., 2005; Couceiro et al., 2006; Yao et al., 2019; Tavakoli et al., 2020; Kaundal et al., 2021). Lower temperatures can enhance plant growth and Hyp accumulation; indeed cooler growth conditions can significantly increase plant biomass by inducing gene expression that favor growth (Brunáková et al., 2015; Yao et al., 2019; Tavakoli et al., 2020). Previous studies have found that *ca.* 750 genes are differentially expressed and 150 genes are involved in plant growth, Hyp biosynthesis and/or environmental responses in *H. perforatum* seedlings at different temperatures (Su et al., 2021). Based on this previous study, St John’s wort expression levels for low-level gene candidates have been quantified by qRT-PCR (real time quantitative PCR), to further probe the mechanism of seedlings performance under a cool temperature. Secretory structures associated with leaf metabolite accumulation were also monitored under reduced temperature conditions.

## 2 Materials and methods

### 2.1 Plant materials

*H. perforatum* seedlings were grown from seed [collected Kangxian county (33°16′20″N, 105°31′50″E; 1050 m a.s.l.) located in Gansu province, China] and acclimated to defined temperatures according to previously published protocols (Yao et al., 2019; Su et al., 2021). Specifically, seeds were successively disinfected with 70% ethanol (v/v) and 0.1% HgCl_2_ (w/v), and then the sterilized seeds were inoculated on Murashige and Skoog (MS) + 20.0 g/L sucrose + 4.0 g/L agar (pH 5.8) and germinated at 22°C (24 h/d photoperiod, white light, 500 µmol·m^−2^·s^−1^ Flux; 50 ± 5% relative humidity). After 25 days of germination at 22°C, seedlings (**Figure S1**) were transplanted to a new MS medium with 0.5 mg/L 1-Naphthylacetic acid (NAA) + 1.0 mg/L 6-Benzylaminopurine (6-BA). After 20 days of growth, half of the healthy seedlings were kept at 22°C and the other half was moved to 15°C (24 h/d photoperiod, white light, 500 µmol·m^−2^·s^−1^ Flux; 50 ± 5% relative humidity) in illuminated incubators (PDX-600A, KunCheng Scientific Instruments Co., Ltd., Shanghai, China). After 20 days growth at 15 and 22°C, the treated seedlings (**Figure S2**) were collected for seedlings dry weight (DW), Hyp quantification, anatomical observation and qRT-PCR validation. Herein, each treatment had 40 flasks with 3 seedlings per flask. Additionally, the criteria for choosing the temperatures is based on our previous findings that the aerial parts biomass and Hyp accumulation in *H. perforatum* are greater at 15°C compared with 22 and 30°C (Yao et al., 2019).

### 2.2 Measurement of chlorophyll and carotenoid contents

Chlorophyll and carotenoid contents were measured according to a previous protocol (Li et al., 2009; Yang et al., 2014). Briefly, fresh whole leaves (0.1 g) were finely ground in 80% acetone (v/v, 5 mL) and centrifuged at 5000 r/min and 4°C for 10 min. The supernatant was diluted to 25 mL with 80% acetone (v/v). Absorbance was taken at 662, 646 and 470 nm using a spectrophotometer (UV-6100, Shanghai, China). The specific calculations are as follows:


$${\text{Chlorophyll a concentration: C}}_{\text{a}}\text{(mg/L)}=12.2A_{662}-2.81A_{646}$$

$${\text{Chlorophyll b concentration: C}}_{\text{b}}\text{(mg/L)}=20.13A_{646}-5.03A_{662}$$

$${\text{Chlorophyll concentration: C}}_{\text{T}}\text{(mg/L)}={\text{C}}_{\text{a}}+{\text{C}}_{\text{b}}$$

$$\text{Carotenoid concentration: C (mg/L})=1000A_{470}-3.27C_{a}-104C_{b})/229$$

Pigment content (mg/g)=(C×V)/M

where “*A_662_*”, “*A_646_*” and “*A_470_*” represent the absorbance at 662, 646 and 470 nm, as well as “*C*”, “*V*” and “*M*” represent the concentration of pigment (mg/L), volume of extract (L) and sample fresh weight (FW, g), respectively.

### 2.3 HPLC quantification of Hyp content

Hyp content was quantified according to previous protocols (Couceiro et al., 2006; Yao et al., 2019). Briefly, air-dried aerial parts of seedlings were finely powdered, samples (0.1 g) were soaked in 95% ethanol (v/v; 20 mL) and agitated in the dark at 22°C for 72 h, and centrifuged at 8000 r/min and 4°C for 10 min. The supernatant was evaporated and concentrated using a vacuum in a rotary evaporator at 60°C, and then the concentrated residue was re-dissolved in methanol (10 mL, chromatography grade). After filtered with a durapore membrane (0.22 μm; Millipore, Sigma, USA), extracts (10 µL) were analyzed at 590 nm by HPLC (Eclipse Plus C18, 250 mm × 4.6 mm, 5 μm; Column temperature 30°C; Agilent 1100 series, Santa Clara, California, USA) and mobile phase with acetonitrile: 50 mmol/L triethylamine (70:30, v/v) at a flow rate of 1.0 mL/min. Hyp content was evaluated on peak area comparison with a reference standard (hypericin, 56690; Sigma Chemical Co., St. Louis, MO, USA). The specific calculations are as follows:


$$\text{Hyp content (mg/g DW})=[Y/Y_{0}\times \Omega \times V]/(M1\times 1000)$$

where “*Ω*”, “*Y_0_*”, “*Y*”, “*V*”, “*M1*” represent the standard concentration of Hyp (µg/mL), standard peak area of Hyp (mAU×s), sample peak area (mAU×s), volume of extract (L) and sample DW (g), respectively.

### 2.4 Leaf micro-structure observations

The middle adaxial leaf of 20 day-old plants was paraffin sectioned based on Li and Zhang, 2016. Briefly, fresh leaves were fixed with a formaldehyde-alcohol-acetic acid (FAA) solution at 4°C for 12 h; fixed samples were then washed with 70% ethanol (v/v) at 22°C for 10 min (thrice), and sequentially dehydrated in 30% ethanol (2 h), 50% (2 h), 70% (12 h), 85% (1 h), 95% (1 h), and 100% (0.5 h) (twice, v/v); dehydrated samples were then sequentially transparentized in the mixture of ethanol and dimethylbenzene (2:1, 1:1, 1:2 and 0:1, v/v) for 30 min. Samples were sequentially immersed in a mixture of dimethylbenzene-paraffin (1:1 and 2:1, v/v) at 56°C for 12 h, and immersed in paraffin at 58°C for 12 h (thrice), and then embedded in paraffin cubes (2 cm); finally, the samples were sliced (7 µm) (KD-2258, Cody, China) and stained (S8020 and F8130, Solarbio, China). Sample imaging was by fluorescence, brightfield and phase contrast microscopy (Revolve RVL-100-G, ECHO, USA).

### 2.5 Leaf ultra-structure analysis

Leaf ultra-structure was observed by transmission electron microscopy (Kornfeld et al., 2007), specific protocols and instrumentation followed previously published literature (Li et al., 2020). Briefly, small pieces (4 mm × 2 mm) of the middle adaxial leaf without mainly veins were firstly immersed into glutaraldehyde (2.5%, v/v) at 4°C for 12 h, then washed with 0.2 M sodium phosphate buffer (pH 7.4) at 22°C for 15 min (thrice), and then fixed with osmium tetroxide (1%, w/v) at 4°C for 5 h; secondly, the fixed samples were washed with the above buffer and then extracted sequentially in 50% ethanol (15 min), 70% (12 h), 80% (15 min), 90% (15 min), 100% (15 min), and acetone (100%) for 15 min (twice, v/v), the mixture agent of acetone and embedding (v/v, 1:1) for 7 h, and embedding medium (Epoxy resin, composed of MNA, EPon-812, DDSA and DMP-30) at 22°C for 12 h; thirdly, the treated leaves were transferred to a embedding plate and immersed in a embedding medium, and then dried sequentially at 35°C for 10 h, 45°C for 12 h and 68°C for 48 h; finally, the embedded samples were sliced (75 nm) with an ultra-microtome (EM UC6, Leica, Germany) and stained with uranyl acetate and lead citrate, and then ultra-structure was observed by a transmission electron microscope (JEM-1230, JEOL Ltd., Japan).

### 2.6 Gene excavation

RNA sequencing was by unigene expression analysis and basic annotation was conducted; 1584 high-level expressed genes with 749 characterized genes and 150 genes involved in plant growth, Hyp biosynthesis and environmental response have been identified with |log_2_(fold-change)| > 1 in previously published article (Su et al., 2021). In this study, low-level genes were identified according to a criteria of 0.2< |log_2_(fold-change)|< 1.0 (Robinson et al., 2009; Love et al., 2014), since low-level genes also play important roles in many biological processes (Maia et al., 2007; Gotor et al., 2010). Differentially Expressed Genes (DEGs) were annotated against the Swiss-Prot database (https://www.uniprot.org/), and 64 candidate genes (**Table S1** and **Table 1**) involved in photosynthesis, energy and Hyp biosynthesis were dug out based on the biological functions.

**Table 1:** Twenty nine genes involved in Hyp biosynthesis in green tissue and dark gland.

Gene name	Swissprot-ID	Protein name	log_2_FC (15°C vs 22°C)
Green tissue (21)			
Glycolysis (10)			
*PFK2*	Q9FIK0	ATP-dependent 6-phosphofructokinase 2	0.22
*PFK3*	Q94AA4	ATP-dependent 6-phosphofructokinase 3	0.23
*PFK4*	Q9FKG3	ATP-dependent 6-phosphofructokinase 4, chloroplastic	0.28
*PFK5*	Q8VYN6	ATP-dependent 6-phosphofructokinase 5, chloroplastic	-0.20
*LTA2*	Q9SQI8	Dihydrolipoyllysine-residue acetyltransferase component 4 of pyruvate dehydrogenase complex, chloroplastic	-0.21
*ENO1*	P42896	Enolase	0.69
*HXK1*	Q9SEK2	Hexokinase-1	0.37
*HXK2*	P93834	Hexokinase-2	0.29
*PFP-ALPHA*	Q41140	Pyrophosphate–fructose 6-phosphate 1-phosphotransferase subunit alpha	0.26
*PFP-BETA*	Q41141	Pyrophosphate–fructose 6-phosphate 1-phosphotransferase subunit beta	0.33
Fatty acid metabolism (11)			
*CUT1*	Q9XF43	3-ketoacyl-CoA synthase 6	0.27
*ACOT13*	Q9NPJ3	Acyl-coenzyme A thioesterase 13	0.23
*Acot9*	Q9R0X4	Acyl-coenzyme A thioesterase 9, mitochondrial	0.26
*LPD1*	Q9M5K3	Dihydrolipoyl dehydrogenase 1, mitochondrial	0.40
*At3g45770*	Q8LCU7	Enoyl-[acyl-carrier-protein] reductase, mitochondrial	-0.49
*MFP2*	Q39659	Glyoxysomal fatty acid beta-oxidation multifunctional protein MFP-a	0.22
*AIM1*	Q9ZPI6	Peroxisomal fatty acid beta-oxidation multifunctional protein AIM1	0.28
*PDH-E1*	O24457	Pyruvate dehydrogenase E1 component subunit alpha-3, chloroplastic	0.27
*ECR*	Q9M2U2	Very-long-chain enoyl-CoA reductase	0.32
*HACD2*	Q2KIP8	Very-long-chain (3R)-3-hydroxyacyl-CoA dehydratase 2	0.28
*KCR1*	Q8L9C4	Very-long-chain 3-oxoacyl-CoA reductase 1	0.72
Dark gland (8)			
*PKSA*	O23674	Type III polyketide synthase A	0.43
*PKSG5*	F1LKH9	Polyketide synthase 5	-0.27
*CHS*	Q9LKP7	Chalcone synthase	0.99
*CHS1*	Q9XGX2	Chalcone synthase 1	-0.64
*FGRAMPH1_01T20223*	Q4I8Q4	Acyl-protein thioesterase 1	0.26
*MALD1*	O50001	Major allergen Pru ar 1	0.63
*STH-2*	P17642	Pathogenesis-related protein STH-2	0.27
At4g20800	Q9SVG7	Berberine bridge enzyme-like 17	0.94

### 2.7 qRT-PCR quantification

Primer sequences of the selected 32 candidate genes (**Table S2**) were designed using a Primer-BLAST tool in NCBI. The coding sequences (CDS) of the 32 genes are shown in **Table S3**. *Actin* (*ACT*) was selected as a reference gene. The extraction of total RNA, synthesis of first-strand cDNA and PCR amplification were performed using RNA kit, RT kit and SuperReal PreMix, respectively. The RNA quality was assessed using an Ultramicro spectrophotometer (Micro Drop, BIO-DL, Shanghai, China) (**Table S4**) and the integrity was evaluated by 1.0% (w/v) agarose gel electrophoresis (**Figure S3**), reverse transcription was performed to generate cDNA on the following protocol: 42°C for 15 min and then 95°C for 3 min, one cycle, PCR amplification was performed on the following protocol: one cycle at 95°C for 15 min, and 35 cycles at 95°C for 10 s, 60°C for 20 s and 72°C for 30 s, and melting curve analysis was performed after a 34 s incubation at 72°C (Yao et al., 2019). The concentrations of cDNA and primer were respectively diluted to 100 ng/μL (2 μL) and 10 μM (1.2 μL) for gene expression analysis. Gene expression was quantified using a LightCycler 96 (Roche, Switzerland). Relative expression level (REL) of gene at 15°C compared with 22°C (Control) was valuated based on a 2*^-△△Ct^* method according to the following formula (Willems et al., 2008):


$$△Ct_{Test\;gene}=Ct_{Test\;gene}-Ct_{Reference\;gene}$$

$$△Ct_{Control\;gene}=Ct_{Control\;gene}-Ct_{Reference\;gene}$$

$$-△△Ct=-(△Ct_{Test\;gene}-△Ct_{Control\;gene})$$

$$\text{REL (Test gene/Control gene)}=2^{-△△Ct}$$

### 2.8 Statistical analysis

Three biological replicates were performed; SPSS 22.0 software was used for a *t*-test analysis with *P*<0.05 for differences.

## 3 Results and discussion

### 3.1 Low temperature increases chlorophyll and carotenoid content

To probe physical and physiological changes in leaves with a change in median growth temperature, a series of growth parameters were monitored. A 1.1- and 1.2-fold increase of chlorophyll (a + b) and carotenoid contents was observed at 15 compared with 22°C, respectively (**Figure S4**). These results were consistent with previous reports that low temperature can significantly increase chlorophyll content, plant growth and subsequently enhance biomass accumulation in comparison with high temperatures (22 and 30°C) (Yao et al., 2019; Su et al., 2021). Five photosynthetic encoded genes (*i.e. psbA*, *psbC*, *ycf4*, *ycf5* and *matK*) were up-regulated at 15°C compared with 22 and 30°C (Yao et al., 2019); and nine genes encoding chlorophyll a-b binding proteins (*i.e. CAB*, *CAB1*, *CAB1B*, *CAB3*, *CAB3C*, *CAB96*, *ELI_PEA*, *OHP2* and *RBCS-C*) were up-regulated at 15°C compared with 22 (Su et al., 2021). The up-regulation of these genes encoding chlorophyll a-b binding, light-induced and light-harvesting complexes proteins indirectly indicate that lower temperatures can improve the accumulation of chlorophyll pigments, which successively enhance photosynthesis and plant growth.

### 3.2 Low temperature increases biomass and Hyp content

**Figure 1:**
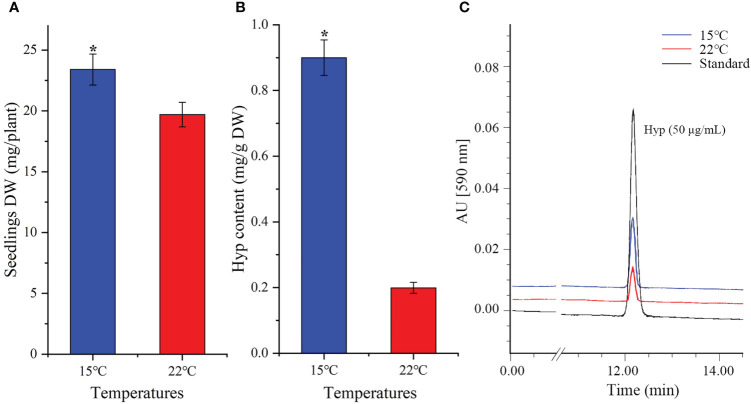
Seedlings DW **(A)**, Hyp content **(B)** and representative chromatograms of HPLC **(C)** in *H perforatum* grown at 15 and 22°C. Values of DW are average with their standard deviations (n = 25), and values of Hyp content are average with their standard deviations (n = 3). The “*” represents a significant difference (*P*< 0.05) between 15 and 22°C.

As shown in **Figure 1**, there were greater biomass and Hyp content at lower temperature, with a 1.2-fold increase of the whole seedlings DW (**Figure 1A**) and 4.5-fold increase of Hyp content in aerial parts at 15 compared with 22°C (**Figure 1B**). The representative chromatograms of reference standard (50 μg/mL, injection volume 10 µL) as well as the extracts (10 mL, injection volume 10 µL) of aerial parts of seedlings at 15 and 22°C were shown in **Figure 1C**. Previous studies on *H. perforatum* have found that cooler temperatures can enhance Hyp accumulation. Specifically, there was a 1.4-fold increase of Hyp content on a DW basis at 15 compared with 22°C after the seedlings treated for 45 days (Yao et al., 2019); a maximum Hyp content on a DW basis at 4 and 8°C compared with 16 and 25°C, with about 10-fold increase at 4 compared with 25°C after the seedlings treated for 7 days (Tavakoli et al., 2020). These findings further demonstrate that Hyp accumulation in *H. perforatum* can be significantly enhanced by cooler temperatures. In fact, extensive experiments have demonstrated that bioactive compounds can be improved at cooler temperatures, such as podophyllotoxin content in *Sinopodophyllum hexandrum* at 15 compared with 22°C (Li et al., 2020), ferulic acid content in *Angelica sinensis* at 15 compared with 22°C (Dong et al., 2022), and total ginsenosides content in *Panax ginseng* at 10 compared with 25°C (Wang et al., 2019).

### 3.3 Low temperature changes leaf cell micro-structure

**Figure 2:**
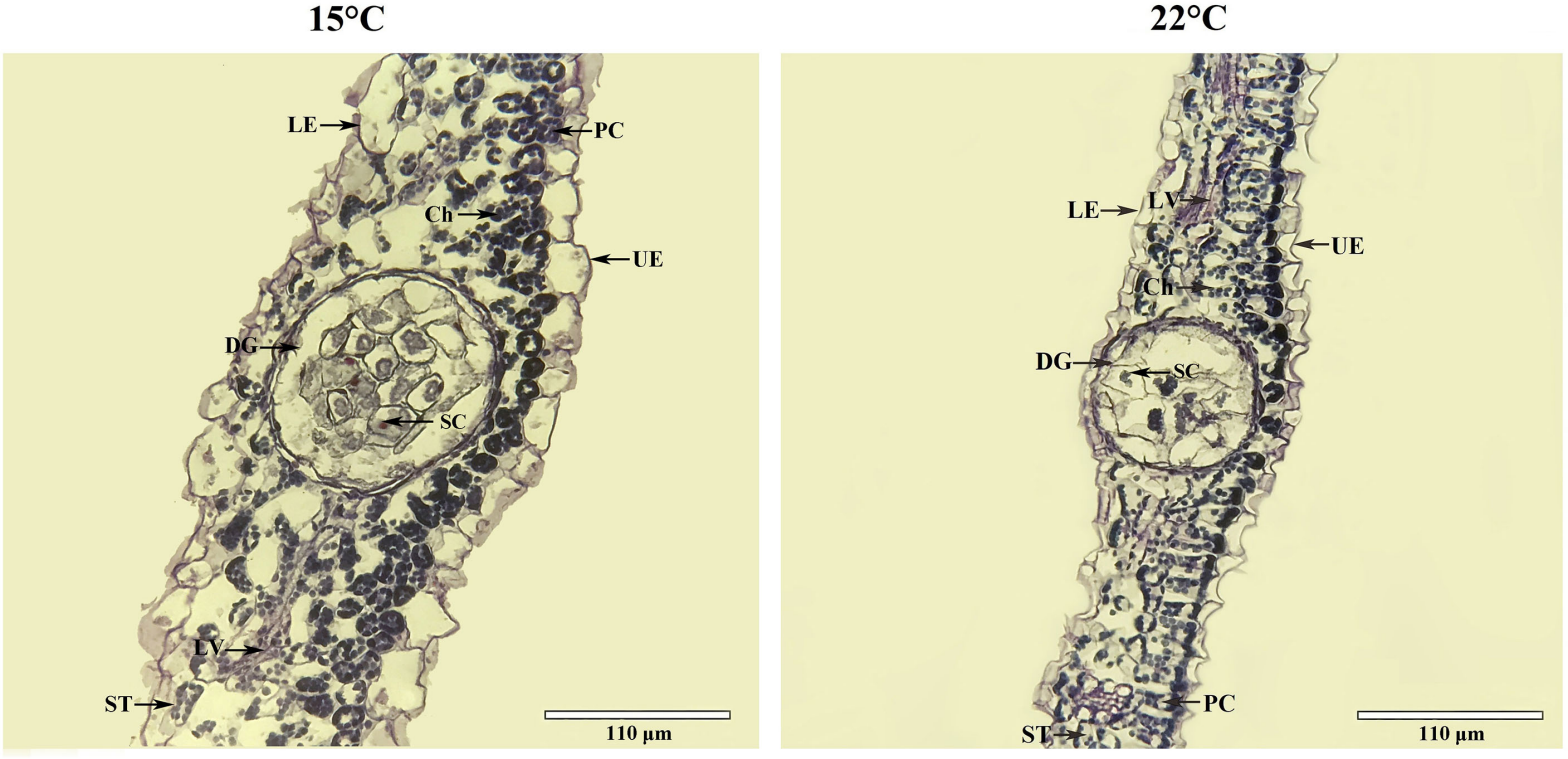
Cross-sectional micro-structure for leaves of seedlings grown at 15 and 22°C. Ch, chloroplast, DG, dark gland, LE, lower epidermis, LV, leaf veins, PC, palisade cell, SC, secretory cell, ST, spongy tissue, UE, upper epidermis.

**Figure 3:**
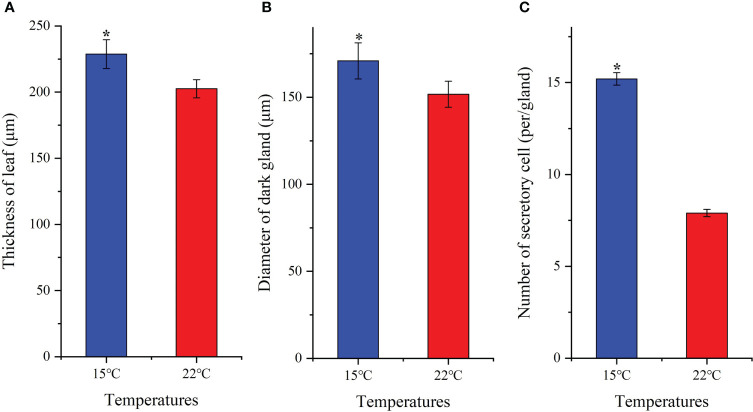
Changes of leaf thickness **(A)**, diameter of dark-gland **(B)** and number of secretory cell **(C)** for seedlings grown at 15 and 22°C. All the values are average with their standard deviations (n = 10). The “*” represents a significant difference (*P*< 0.05) between 15 and 22°C.

Leaf tissue structure alterations [*i.e.* lower epidermis (LE), upper epidermis (UE), palisade cell (PC), spongy tissue (ST) and leaf veins (LV)] as well as organelle density [*i.e.* chloroplast (Ch), dark gland (DG) and secretory cell (SC)] were observed based on growing temperatures (**Figure 2**). A 1.1-fold increase in leaf thickness was detected (**Figure 3A**). Previous studies have found that *H. perforatum* leaf morphology (*e.g.* leaf length/width, stem height and DG) are significantly affected by species, geographic, light and temperature conditions (Briskin and Gawienowski, 2001; Walker et al., 2001; Cirak et al., 2007; Stoyanova-Koleva et al., 2015; Su et al., 2021). Since Hyp biosynthesis occurs in dark glands (DG) and secretory cells (SC) is associated with Hyp accumulation (Lv and Hu, 2001; Zobayed et al., 2006; Kornfeld et al., 2007; Rizzo et al., 2019), these organelles were monitored. Increases in DG size and SC number of 1.2-fold and 1.9-fold, respectively for plants growing at the lower temperature was observed (**Figures 3B, C**). Studies with *Camellia oleifera* grown at 15 compared with 16°C exhibited an increased leaf thickness, of 1.2-fold (Hu et al., 2016). Previous studies on *H. perforatum* have found that the number of DG is more at 15°C compared with 22°C (Su et al., 2021). Thus, larger size of DG and more number of SC in this study further confirm previous studies that higher Hyp accumulates to a greater level at 15°C than 22°C (Su et al., 2021).

### 3.4 Low temperature changes leaf cell ultra-structure

**Figure 4:**
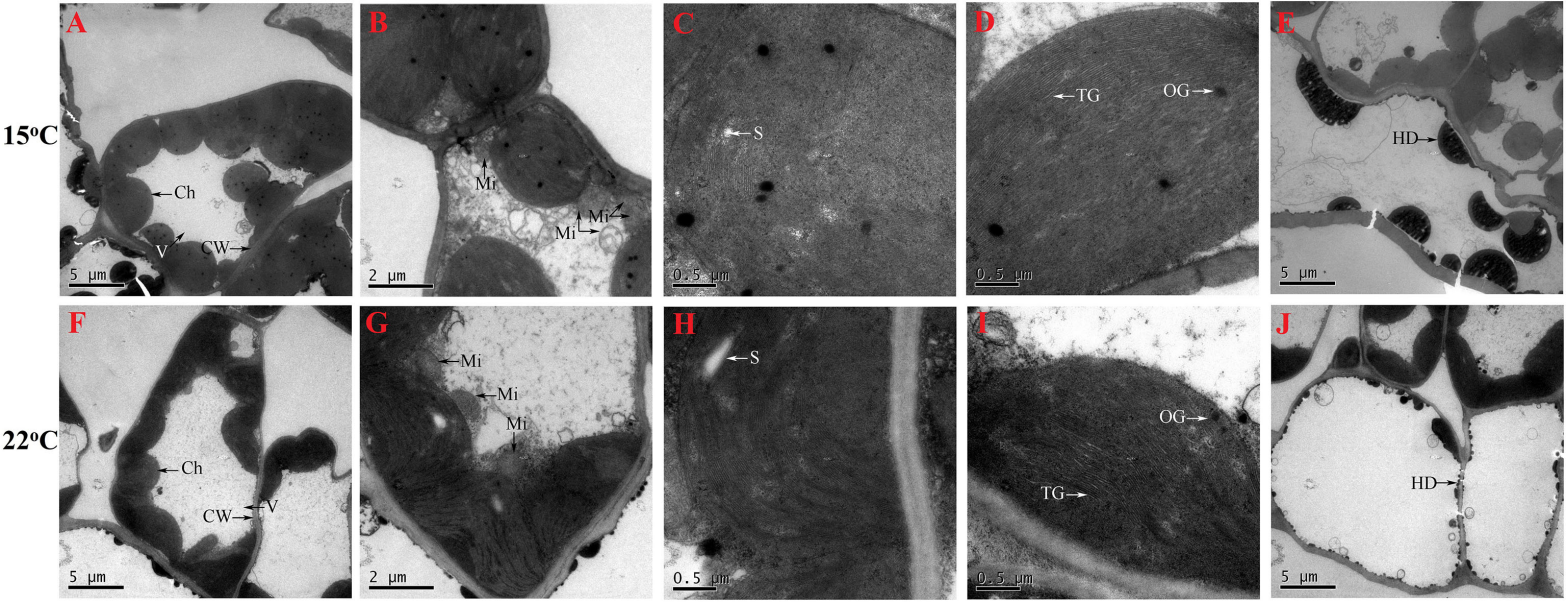
Cell ultra-structure for seedlings grown at 15 **(A–E)** and 22°C **(F–J)**. Ch, chloroplast, CW, cell wall; Mi, mitochondria; HD, hemispherical droplets; OG, osmiophilic granule; S, starch grain; TM, thylakoid grana; V, vacuole.

Vacuole (V) occupied most of the space of whole cell, and chloroplasts (Ch) were near to the cell wall (CW) (**Figure 4A, F**); mitochondria (Mi) were near to the Ch (**Figures 4B, G**); starch grains (S) (**Figures 4C, H**), thylakoid grana (TG) and osmiophilic granules (OG) (**Figures 4D, I**) presented in the Ch; and hemispherical droplets (HD) (**Figures 4E, J**) appeared in the epidermal cell. Based on the observations (n=10), the number of Ch, Mi, S, TG and OG appeared to be greater and the size of HD was significantly larger at 15°C compared with 22°C (**Figure 4**). The number of Ch, Mi, S, TG and OG affected by abiotic stresses such as temperatures has been observed in other plants (Zhang et al., 2005; Li et al., 2020). Here, an increase in the number of Ch, Mi, S, TG and OG may be a low-temperature response for energy acquisition and utilization, since previously studying *H. perforatum* have reported that cooler temperature can enhance plant growth (Yao et al., 2019; Su et al., 2021). The HD, which seems to adhere to membranes or is somehow trapped in a hemispherical shape, may be associated with Hyp biosynthesis (Kornfeld et al., 2007). Here, an increase in the size of HD may play a certain role in enhancing Hyp biosynthesis at lower temperature.

### 3.5 Low temperature regulates gene expression related to photosynthesis and energy

**Figure 5:**
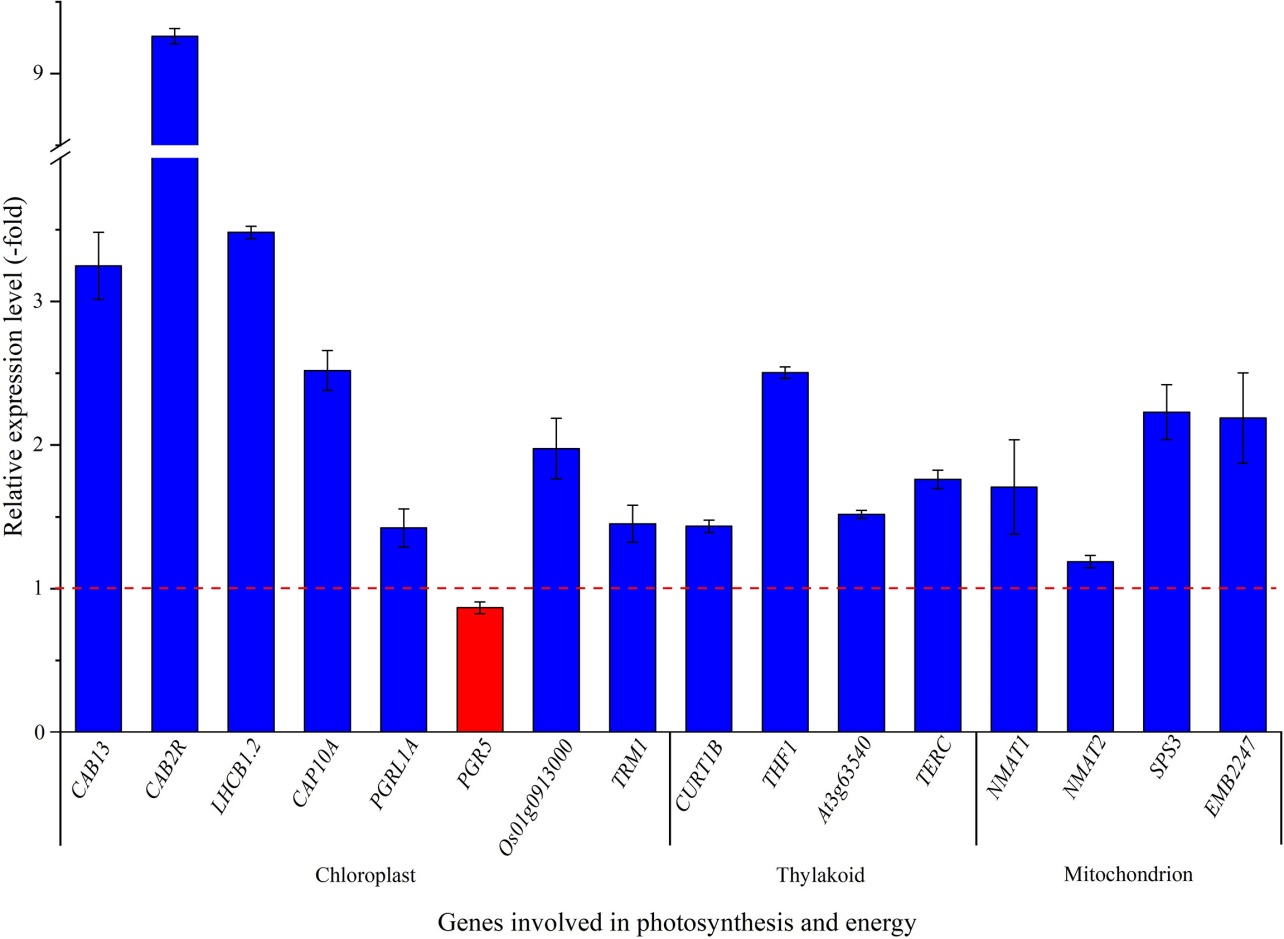
The expression level of genes involved in chloroplast, thylakoid and mitochondrion for seedlings grown at 15 versus 22°C, as determined by qRT-PCR (n=3). Column highlighted in blue represents gene up-regulation and red represents gene down-regulation. The red dotted line in the image differentiates up-regulation (>1) and down-regulation (<1) at 15°C compared with 22°C (Control), respectively.

Thirty-five genes related to photosynthesis and energy (Taiz and Zeiger, 2010) was observed to be differentially regulated with temperature (**Table S1**), and 16 genes were selected to validate the expression levels. Fifteen genes (*i.e. CAB13*, *CAB2R*, *LHCB1.2*, *CAP10A*, *PGRL1A*, *Os01g0913000*, *TRM1*, *CURT1B*, *THF1*, *At3g63540*, *TERC*, *NMAT1*, *NMAT2*, *SPS3* and *EMB2247*) presented a 1.4 to 9.3-fold up-regulation, while *PGR5* presented a 0.9-fold down-regulation at 15°C compared with 22°C (**Figure 5**). In previous studies, 12 high-level genes (*i.e.* six *CABs*, *ELI_PEA*, *ELIP2*, *OHP2*, *RBCS-C*, *RCA* and *RBCS-8B*) related to photosynthesis were differentially expressed at 15°C compared with 22°C (Su et al., 2021).

For the specific biological functions of the selected 8 genes related to chloroplast, *CAB13*, *CAB2R*, *LHCB1.2* and *CAP10A* encode light-harvesting chlorophyll a-b binding proteins (LHCs) that functions as a light receptor and play indispensable roles in capturing and delivering excitation energy to photo systems (Zou et al., 2020), *PGRL1A* and *PGR5* are involved in electron flow (Munekage et al., 2002; Hertle et al., 2013), *Os01g0913000* and *TRM1* are involved in various redox reactions (Capitani et al., 2000; Glauser et al., 2004). For selected genes associated with the thylakoid membrane, *CURT1B* determines thylakoid architecture by inducing membrane curvature (Armbruster et al., 2013), *THF1* is required for the formation of mature thylakoid stacks from the normal vesicles (Wang et al., 2004), *At3g63540* is involved in the folding and proteolysis of thylakoid proteins (Peltier et al., 2002), and *TERC* is involved in the thylakoid formation (Kwon and Cho, 2008; Schneider et al., 2014). For these four genes related to mitochondrion, *NMAT1* and *NMAT2* are required for mitochondrial biogenesis and the regulation of fundamental metabolic pathways during early developmental stages (Nakagawa and Sakurai, 2006), *SPS3* is involved in the ubiquinone-9 biosynthesis from solanesyl diphosphate (Ducluzeau et al., 2012), and *EMB2247* is involved in the formation of carbon-oxygen bonds in aminoacyl-tRNA (Berg et al., 2005). The up-regulation of these genes involved in photosynthesis and energy will confer *H. perforatum* seedlings to grow robust and adapt cooler temperatures compared with higher temperatures.

### 3.6 Low temperature regulates gene expression related to Hyp biosynthesis

#### 3.6.1 Mapping genes related to Hyp biosynthesis

**Figure 6:**
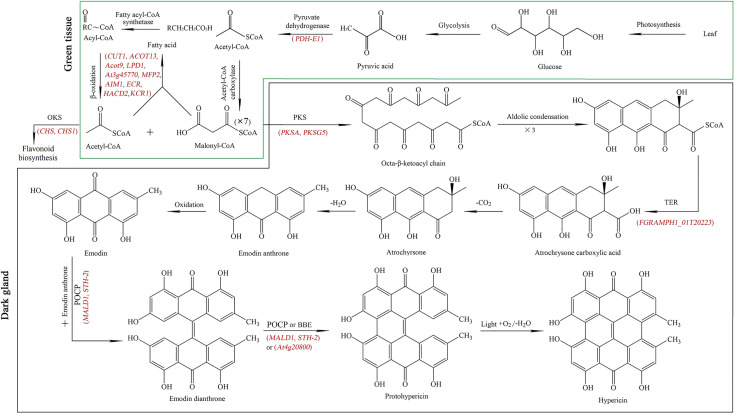
Key genes (red color) mapped in the Hyp biosynthetic pathway from glucose to acetyl- and malonyl-CoA in green tissue (green frame), and from acetyl- and malonyl-CoA to Hyp in dark gland (dark frame). PKS: polyketide synthase, OKS: octaketide synthase, TER: thioesterase, POCP: phenoloxidative coupling protein, BBE: berberine bridge enzyme. The Hyp biosynthetic pathway concludes from previous literatures (Zobayed et al., 2006; Rizzo et al., 2019; Rizzo et al., 2020; Su et al., 2021).

While previous studies have revealed that Hyp is biosynthesized in two separated tissues including: (1) green tissue from glucose to acetyl- and malonyl-CoA, and (2) dark gland from octa-β-ketoacyl chain to Hyp (Zobayed et al., 2006; Rizzo et al., 2019; Yao et al., 2019; Rizzo et al., 2020; Su et al., 2021), Hyp biosynthetic has not been fully elucidated and some genes have still not been identified. In this study, twenty-nine genes participating in Hyp biosynthesis were identified, with 21 genes in green tissue including in glycolysis (10 genes, 8 up-regulated and 2 down-regulated) and fatty acid metabolism (11 genes, 10 up-regulated and 1 down-regulated), and 8 genes in dark gland (6 up-regulated and 2 down-regulated) (**Table 1**). In the green tissue, acetyl-CoA is formed through photosynthesis, glycolysis (*i.e. PFK2*, *PFK3*, *PFK4*, *PFK5*, *LTA2*, *ENO1*, *HXK1*, *HXK2*, *PFP-ALPHA* and *PFP-BETA*) and pyruvate dehydrogenase (*i.e. PDH-E1*), malonyl-CoA is formed from acetyl-CoA *via* acetyl-CoA carboxylase, meanwhile, fatty acid metabolism (*i.e. CUT1*, *ACOT13*, *Acot9*, *LPD1*, *At3g45770*, *MFP2*, *AIM1*, *ECR*, *HACD2* and *KCR1*) is involved in the biosynthesis of the acetyl-CoA and malonyl-CoA. For the biosynthetic pathway in the dark gland, an octa-β-ketoacyl chain is formed with one acetyl-CoA and seven malonyl-CoAs by the PKS (*i.e. PKSA* and *PKSG5*), emodin anthrone is formed through a series of aldolic condensation, thioesterase (TER) (*i.e. FGRAMPH1_01T20223*), decarboxylic and dehydration reactions, then emodin dianthrone is produced by the oxidation of emodin anthrone as well as the coupling of emodin with emodin anthrone by the phenoloxidative coupling protein (POCP) (*i.e. MALD1* and *STH-2*); finally, Hyp is generated by POCP or berberine bridge enzyme (BBE) (*i.e. At4g20800*) as well as light, oxidation and dehydration reactions (**Figure 6**). While there is a competitive relationship between the PKS and octaketide synthase (OKS) (*i.e. CHS* and *CHS1*) in this study, because the OKS can catalyze the 4-coumaroyl-CoA and malonyl-CoA precursors into flavonoids (*i.e.* 2’,4,4’,6’-tetrahydroxychalcone) (Ferrer et al., 1999).

#### 3.6.2 Expression level of genes in green tissue

**Figure 7:**
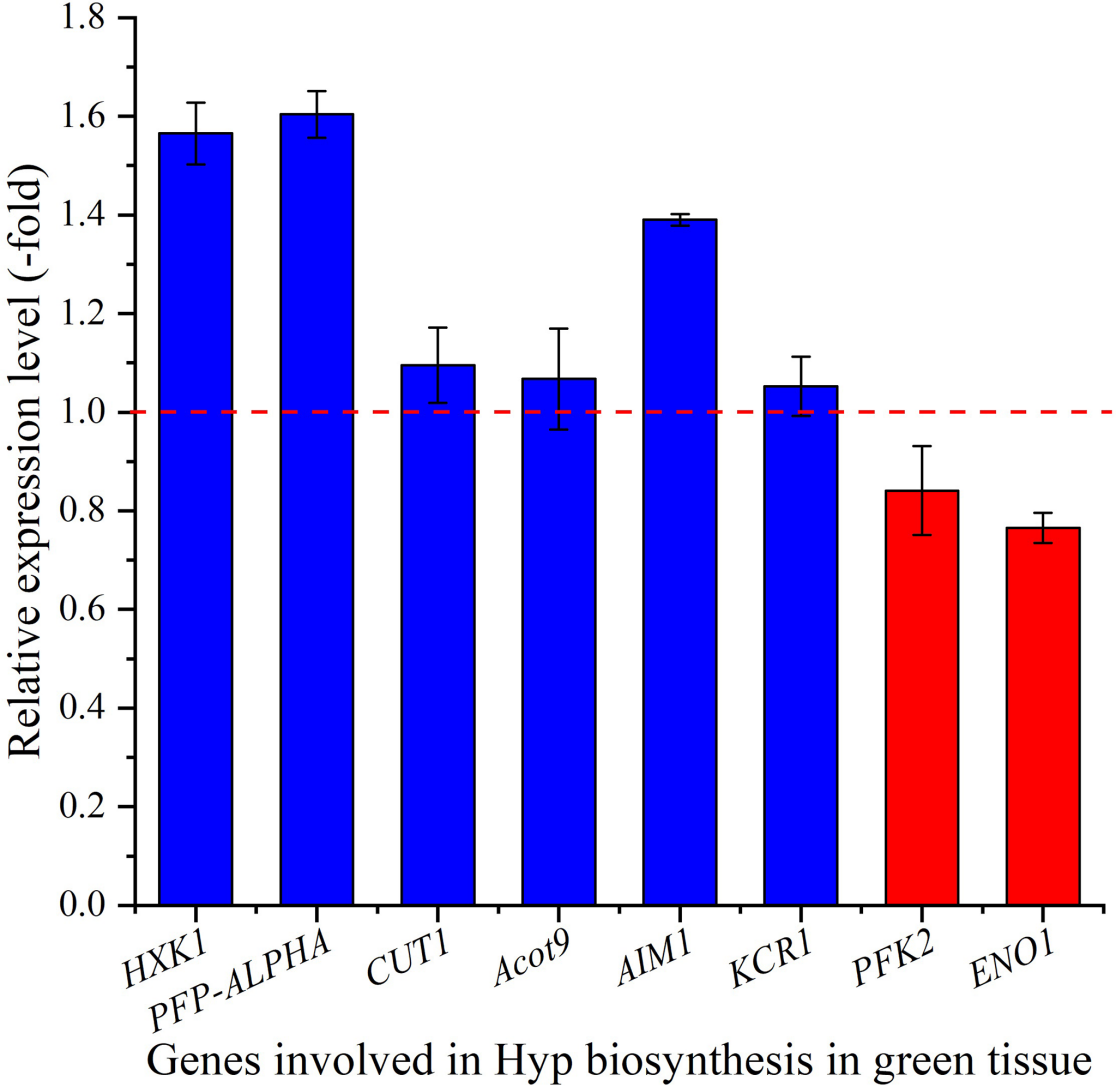
The expression level of genes involved in Hyp biosynthesis in green tissue for seedlings grown at 15 versus 22°C, as determined by qRT-PCR (n=3). Column highlighted in blue represents gene up-regulation and red represents gene down-regulation. The red dotted line in the image differentiates up-regulation (>1) and down-regulation (<1) at 15°C compared with 22°C (Control), respectively.

The relative expression of selected genes in the green tissue were observed to be differentially regulated, with up-regulation of 1.6-, 1.6-, 1.1-, 1.1-, 1.4- and 1.1-fold for the 6 genes *HXK1*, *PFP-ALPHA*, *CUT1*, *Acot9*, *AIM1* and *KCR1*, while down-regulation of 0.8- and 0.8-fold for the 2 genes *PFK2* and *ENO1*, respectively at 15°C compared with 22°C (**Figure 7**). For selected genes involved in glycolysis, *PFK2* is involved in the formation of fructose 1,6-bisphosphate by phosphorylating D-fructose 6-phosphate (Mustroph et al., 2007), *ENO1* is involved in catalyzing the formation of phosphoenolpyruvate from 2-phosphoglycerate (Allen and Whitman, 2021), *HXK1* and *PFP-ALPHA* are involved in the formation of D-glyceraldehyde 3-phosphate and glycerone phosphate (Todd et al., 1995; Giese et al., 2005). For genes involved in fatty-acid metabolism, *CUT1* participates in both decarbonylation and acyl-reduction wax synthesis pathways (Fiebig et al., 2000), *Acot9* is involved in the formation of free fatty acid and coenzyme A by hydrolyzing of acyl-CoAs (Poupon et al., 1999), *AIM1* is involved in the peroxisomal beta-oxidation pathway for the biosynthesis of benzoic acid (Bussell et al., 2014), and *KCR1* is responsible for the first reduction step in very long-chain fatty acids synthesis (Beaudoin et al., 2009). The up-regulation of these genes in green tissue at cooler temperature is likely to provide abundant acetyl-CoA and malonyl-CoA as precursors for downstream Hyp biosynthesis.

#### 3.6.3 Expression level of genes in dark gland

**Figure 8:**
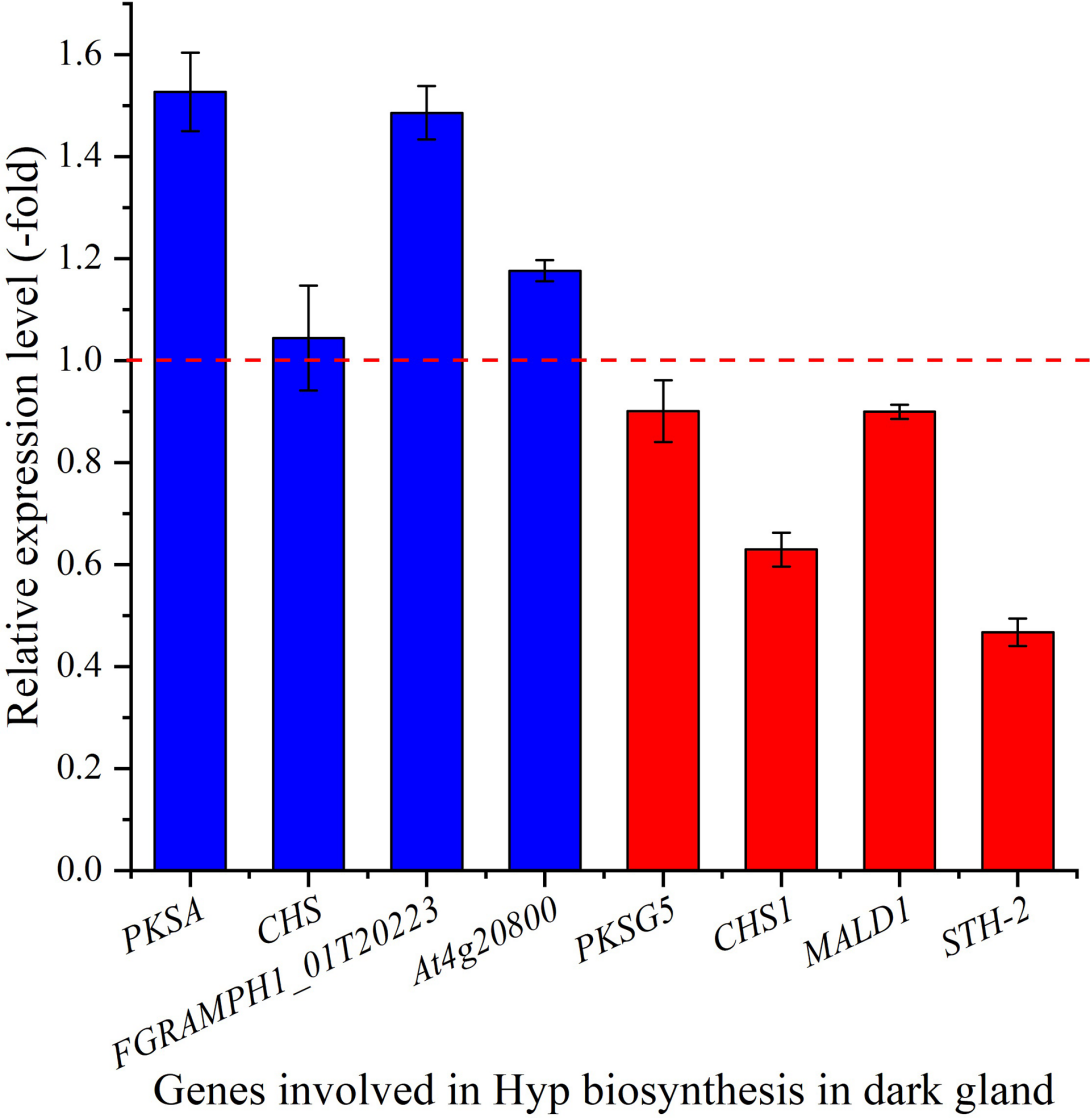
The expression level of genes involved in Hyp biosynthesis in dark gland for seedlings grown at 15 versus 22°C, as determined by qRT-PCR (n=3). Column highlighted in blue represents gene up-regulation and red represents gene down-regulation. The red dotted line in the image differentiates up-regulation (>1) and down-regulation (<1) at 15°C compared with 22°C (Control), respectively.

The relative expression of selected genes in dark glands were also observed to be differentially regulated, with up-regulation of 1.5-, 1.5- and 1.2-fold for the 3 genes *PKSA*, *FGRAMPH1_01T20223* and *At4g20800*, while down-regulation of 0.9-, 0.9- and 0.5-fold for the genes *PKSG5*, *MALD1* and *STH-2*, respectively at 15°C compared with 22°C (**Figure 8**). Both *PKSA* and *PKSG5* encode polyketide synthase that are involved in the condensation of malonyl-CoA units (Mizuuchi et al., 2008; Flores-Sanchez et al., 2010), *FGRAMPH1_01T20223* is predicted to encode TER1 that participates in the formation of emodin anthrone (Kong et al., 2013), *MALD1* and *STH-2* are predicted to encode POCP, and *At4g20800* encodes BBE-like 17 that catalyzes the oxidation of aromatic allylic alcohols (Daniel et al., 2015). The up-regulation of these genes (*PKSA*, *FGRAMPH1_01T20223* and *At4g20800*) in dark glands at a cooler temperature is predicted to play a role in inducing Hyp biosynthesis and accumulation. In this study, the two *CHS* and *CHS1* genes are not up-regulated, and the significant down-regulation (0.63-fold) of *CHS1* might indicate that the reduced temperatures negatively affect phenylpropanoid biosynthesis. If this effect is directly connected to the up-regulation of the Hyp biosynthetic pathway *via* redirecting the pool of 4-coumaroyl-CoA and malonyl-CoA precursors remains to be established. This will require quantitative phenolic profiling by LC-MS combined with flux analysis, but is beyond the scope of this manuscript.

## 4 Conclusions

In *Hypericum perforatum*, low temperature changes cell structure (*e.g.* dark gland, secretory cell and hemispherical droplet) associated with regulating plant growth and gene expression (*e.g. BBE*, *POCP* and *TER1*) associated with Hyp biosynthesis in leaf green tissue and dark gland. These findings not only further confirm that low temperature enhances plant growth and Hyp biosynthesis (Yao et al., 2019; Tavakoli et al., 2020), but also complement previous transcriptomic analysis (Su et al., 2021). Moreover, these findings will provide useful references for guiding *H. perforatum* cultivation in field or green house, cell and tissue culture, and revealing the mechanism of Hyp biosynthesis to increase Hyp accumulation.

## Data availability statement

The datasets presented in this study can be found in online repositories. The names of the repository/repositories and accession number(s) can be found in the article/**Supplementary Material**.

## Author contributions

HS: data curation and investigation. LJ: Resourses. ML: conceptualization, project administration and writing—original draft. PP: writing—review and editing. All authors contributed to the article and approved the submitted version.

## Funding

This research was funded by State Key Laboratory of Aridland Crop Science/Gansu Agricultural University (GSCS-2021-Z03), Assurance Project of Ecological Planting and Quality of Daodi Herbs (202103003).

## Conflict of interest

The authors declare that the research was conducted in the absence of any commercial or financial relationships that could be construed as a potential conflict of interest.

## Publisher’s note

All claims expressed in this article are solely those of the authors and do not necessarily represent those of their affiliated organizations, or those of the publisher, the editors and the reviewers. Any product that may be evaluated in this article, or claim that may be made by its manufacturer, is not guaranteed or endorsed by the publisher.
