# Supplementary material for: Low temperature modifies seedling leaf anatomy and gene expression in Hypericum perforatum
Source: Front Plant Sci. 2022 Sep 27;13:1020857. doi: 10.3389/fpls.2022.1020857 (PMC9552896; doi:10.3389/fpls.2022.1020857)
Supplement: Supplementary file 1 [file DataSheet_1.docx]

**Supplementary Materials**


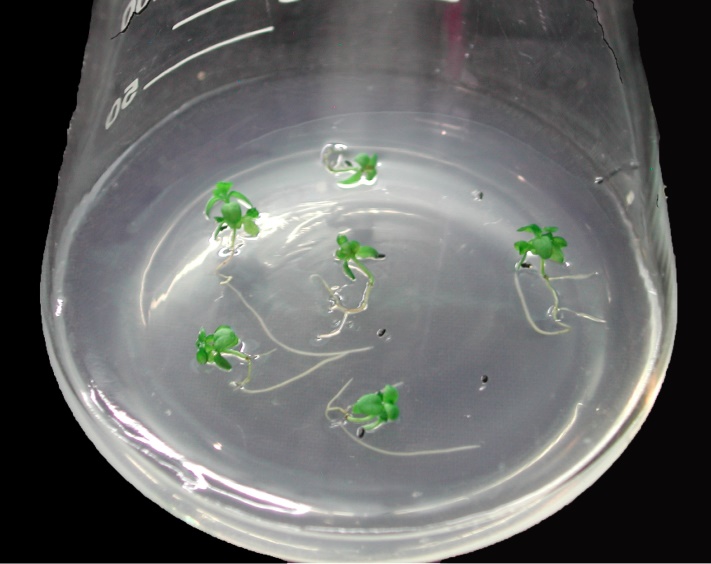


**Fig. S1** Growth characteristics of germinated seeds after 25 days.


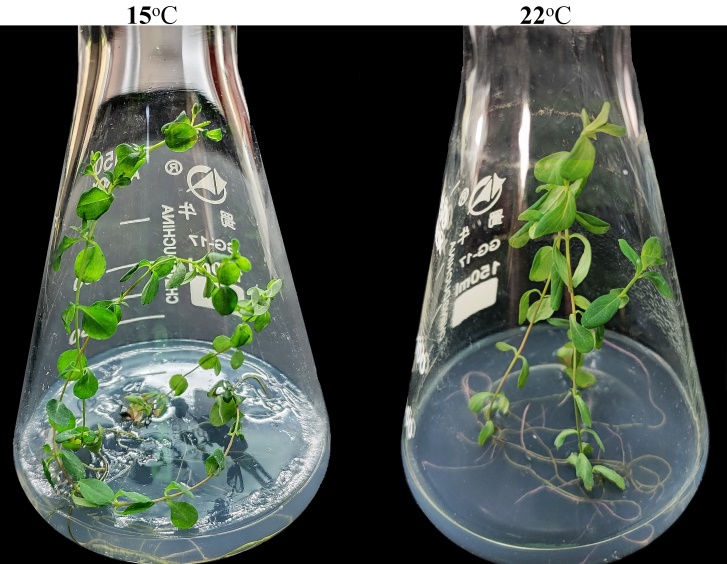


**Fig. S2** Seedlings growth characteristics of *H. perforatum* treated at 15 and 22℃ after 20 days.


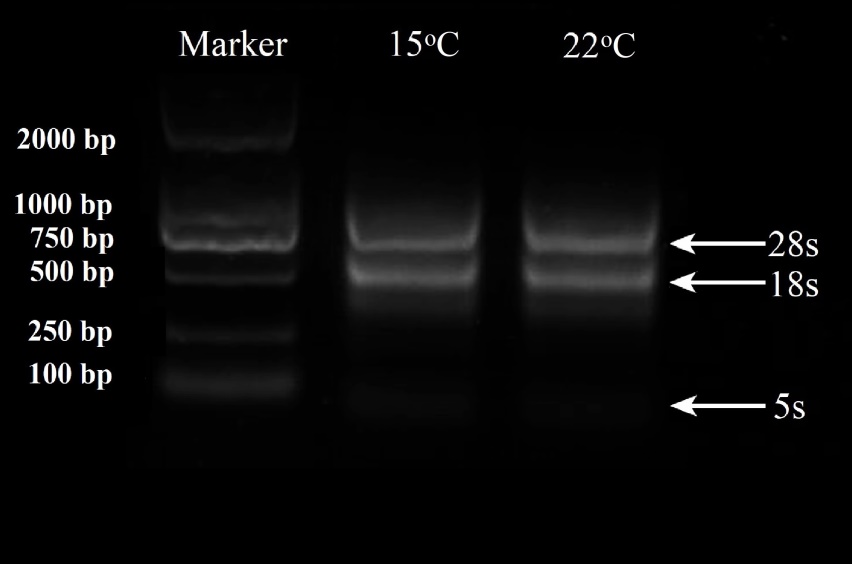


**Fig. S3** The quality of total RNA at 15 and 22◦C was evaluated by 1.0 % agarose gel electrophoresis.


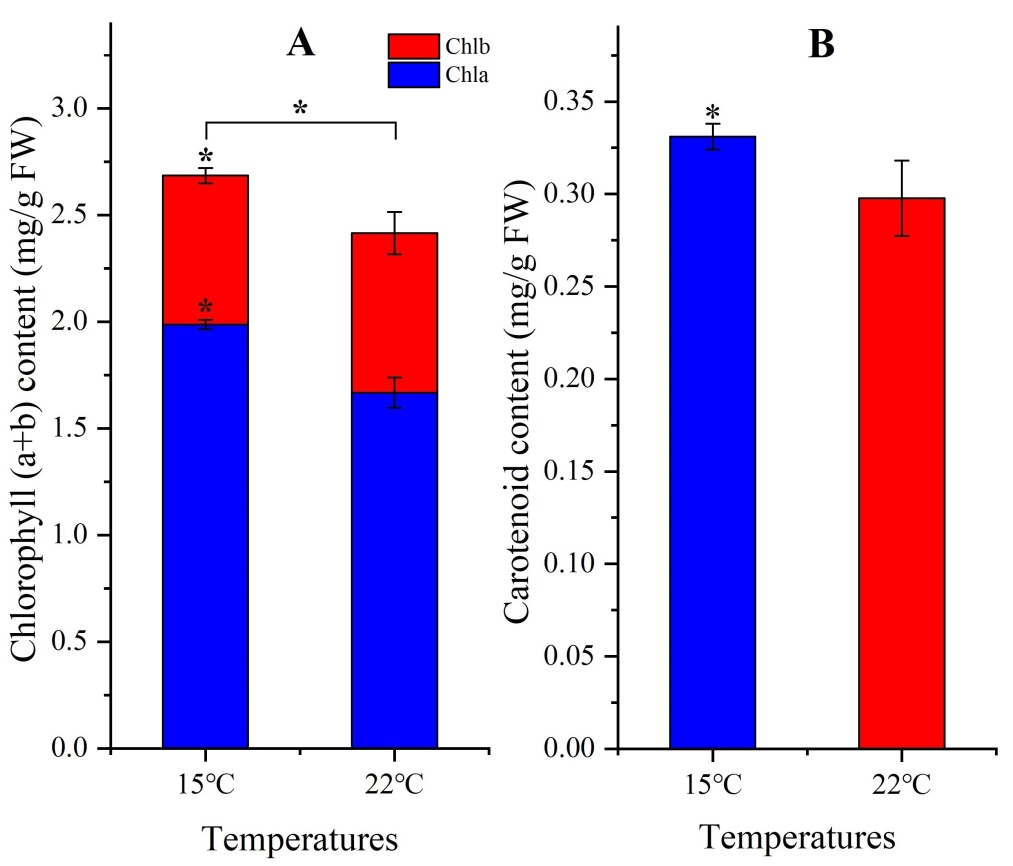


Fig. S4 Chlorophyll (a+b) and carotenoid contents in *H. perforatum* seedlings grown at 15 and 22°C. Values are average with their standard deviations (n = 3). The “*” represents a significant difference (*P* < 0.05) between 15 and 22°C.

**Table S1** Thirty five genes involved in photosynthesis and energy.

| Gene name | Swissprot-ID | Protein name | log_2_FC (15°C vs 22°C) |
| --- | --- | --- | --- |
| Chloroplast (14) | | |  |
| *CAB2R* | P12331 | Chlorophyll a-b binding protein 2, chloroplastic | 0.79 |
| *LHCB1.2* | Q8VZ87 | Chlorophyll a-b binding protein 3, chloroplastic | 0.68 |
| *CAB13* | P27489 | Chlorophyll a-b binding protein 13, chloroplastic | 0.59 |
| *CAP10A* | P27524 | Chlorophyll a-b binding protein CP24 10A, chloroplastic | 0.32 |
| *petC* | P08980 | Cytochrome b6-f complex iron-sulfur subunit, chloroplastic | 0.29 |
| *GSA1* | P45621 | Glutamate-1-semialdehyde 2,1-aminomutase, chloroplastic | 0.44 |
| *CHLH* | Q9FNB0 | Magnesium-chelatase subunit ChlH, chloroplastic | 0.43 |
| *PGRL1A* | Q8H112 | PGR5-like protein 1A, chloroplastic | 0.38 |
| *At1g32060* | P27774 | Phosphoribulokinase, chloroplastic | 0.70 |
| *PSAF* | Q9SHE8 | Photosystem I reaction center subunit III, chloroplastic | 0.37 |
| *CCD4* | O49675 | Probable carotenoid cleavage dioxygenase 4, chloroplastic | 0.35 |
| *PGR5* | Q9SL05 | Protein PROTON GRADIENT REGULATION 5, chloroplastic | 0.89 |
| *Os01g0913000* | P09856 | Thioredoxin F-type, chloroplastic | 0.81 |
| *TRM1* | P07591 | Thioredoxin M-type, chloroplastic | 0.70 |
| Thylakoid (14) | | |  |
| *TPP2* | Q9M9Z2 | Probable thylakoidal processing peptidase 2, chloroplastic | 0.28 |
| *CURT1A* | O04616 | Protein CURVATURE THYLAKOID 1A, chloroplastic | 0.34 |
| *CURT1B* | Q8LCA1 | Protein CURVATURE THYLAKOID 1B, chloroplastic | 0.54 |
| *CURT1D* | Q8LDD3 | Protein CURVATURE THYLAKOID 1D, chloroplastic | 0.25 |
| *THF1* | Q7XAB8 | Protein THYLAKOID FORMATION1, chloroplastic | 0.69 |
| *At2g44920* | O22160 | Thylakoid lumenal 15 kDa protein 1, chloroplastic | -0.31 |
| *At5g52970* | Q9LVV5 | Thylakoid lumenal 15.0 kDa protein 2, chloroplastic | -0.21 |
| *At4g02530* | O22773 | Thylakoid lumenal 16.5 kDa protein, chloroplastic | -0.27 |
| *TL17* | P81760 | Thylakoid lumenal 17.4 kDa protein, chloroplastic | 0.41 |
| *At4g24930* | Q9SW33 | Thylakoid lumenal 17.9 kDa protein, chloroplastic | 0.21 |
| *At3g63540* | P82658 | Thylakoid lumenal 19 kDa protein, chloroplastic | 0.37 |
| *TL20.3* | Q8H1Q1 | Thylakoid lumenal protein TL20.3, chloroplastic | 0.23 |
| *slr0575* | Q55403 | Thylakoid membrane protein slr0575 | 0.39 |
| *TERC* | F4JZG9 | Thylakoid membrane protein TERC, chloroplastic | 0.25 |
| Mitochondrion (7) | | |  |
| *NMAT1* | Q9C8R8 | Nuclear intron maturase 1, mitochondrial | 0.32 |
| *NMAT2* | Q9FJR9 | Nuclear intron maturase 2, mitochondrial | 0.42 |
| *PTPMT1* | Q86BN8 | Phosphatidylglycerophosphatase and protein-tyrosine phosphatase 1 | -0.23 |
| *SPS3* | Q5HZ00 | Solanesyl diphosphate synthase 3, chloroplastic/mitochondrial | 0.88 |
| *SDH2-1* | Q8LBZ7 | Succinate dehydrogenase [ubiquinone] iron-sulfur subunit 1, mitochondrial | 0.22 |
| *UNG* | Q9LIH6 | Uracil-DNA glycosylase, mitochondrial | -0.25 |
| *EMB2247* | F4KE63 | Valine--tRNA ligase, chloroplastic/mitochondrial 2 | 0.34 |

**Table S2** Sequences of primer used in qRT-PCR validation.

| Gene name | Accession ID | Primer sequences (5' to 3') | Amplicon size (bp) |  |
| --- | --- | --- | --- | --- |
| *ACT* | CP002685.1 | Forward: ATCCTCCGTCTTGACCTTGC | 104 |  |
|  |  | Reverse: ACGATTTCCCGTTCTGCTGT |  |  |
| Chloroplast (8) | | | |  |
| *CAB13* | Unigene0011257 | Forward: GGTGACTATGGCTGGGACAC | 183 |  |
|  |  | Reverse: ACCAGCCTTGAACCATACCG |  |  |
| *CAB2R* | Unigene0039379 | Forward: CGGCTGACCCAGAGACATTC | 188 |  |
|  |  | Reverse: CCCCAAGTAGTCCAACCCAC |  |  |
| *LHCB1.2* | Unigene0002933 | Forward: GGAAGACCGCCTCCAAGAAG | 175 |  |
|  |  | Reverse: TTAGCGAATGTCTCGGGGTC |  |  |
| *CAP10A* | Unigene0037069 | Forward: ACTATGGTTTCGACCCGCTG | 118 |  |
|  |  | Reverse: CCTACGAAGATCCCGAGCAC |  |  |
| *PGRL1A* | Unigene0043218 | Forward: TATTTGGGTGGGCCAACTGC | 107 |  |
|  |  | Reverse: CCGTTGACATGAAACGGCAC |  |  |
| *PGR5* | Unigene0044620 | Forward: TTGCACCTGTTGTTGTCGTC | 125 |  |
|  |  | Reverse: ATCTGCTCCGATCGACTTGC |  |  |
| *Os01g0913000* | Unigene0033391 | Forward: AAGCTTGTCAGTGCCGTGAAC | 131 |  |
|  |  | Reverse: TTGACGATAGGCCAGAACGTG |  |  |
| *TRM1* | Unigene0011736 | Forward: CCCCGTGATTGATGAATTGGC | 125 |  |
|  |  | Reverse: AGAAGAGCACGGTCGGAATG |  |  |
| Thylakoid (4) | | | | |
| *CURT1B* | Unigene0044321 | Forward: TCGAAAGATTGCTCGCAGTG | 139 |  |
|  |  | Reverse: TGAGCCCACTGCATACTTGTC |  |  |
| *THF1* | Unigene0005651 | Forward: CAGTTGCTTCCGTGTCGTTC | 166 |  |
|  |  | Reverse: GAGGAACCAGCTCCACTACG |  |  |
| *At3g63540* | Unigene0012158 | Forward: TGTACGATGTCCCTGAAGGC | 132 |  |
|  |  | Reverse: TCCCGGATAGGTACGTCAGG |  |  |
| *TERC* | Unigene0038881 | Forward: TCGGCTTCGAAACTATCCCG | 135 |  |
|  |  | Reverse: TCCATCTTATCAACGGGGCG |  |  |
| Mitochondrion (4) | | | | |
| *NMAT1* | Unigene0005032 | Forward: GGTGATGGAGCCCTACTTCG | 123 |  |
|  |  | Reverse: AAAAACCACAGGTACCCCGC |  |  |
| *NMAT2* | Unigene0043197 | Forward: TTGCGGTGTTCTTAGCCCTC | 177 |  |
|  |  | Reverse: CCGCTCGTGGGAACAAATTC |  |  |
| *SPS3* | Unigene0034363 | Forward: TTGACGCCAAGGAACCACG | 116 |  |
|  |  | R Reverse: AGCAGATGAGGTAAGCTTTGGAG |  |  |
| *EMB2247* | Unigene0009125 | Forward: GGTCTCGACCGATATGAGGC | 102 |  |
|  |  | Reverse: GGATCTGGGCACTCGTAAGG |  |  |
| Glycolysis (4) | | | |  |
| *PFK2* | Unigene0034432 | Forward: CGGGGCTGTGGAGATATTCG | 183 |  |
|  |  | Reverse: TTAACTGCACTCTCGGCCTC |  |  |
| *ENO1* | Unigene0004025 | Forward: GGGACGGAGGCAAGGATTAC | 116 |  |
|  |  | Reverse: ATCGATAGCGGTCTGCTCAC |  |  |
| *HXK1* | Unigene0043512 | Forward: ACCCTGGTGAGCAGATCTTTG | 164 |  |
|  |  | Reverse: AGCTGACATATCGGGCGTTC |  |  |
| *PFP-ALPHA* | Unigene0002246 | Forward: CTTGCAGCTGGATTGAACGG | 141 |  |
|  |  | Reverse: ACTAGCACTTGCACCTGGAC |  |  |
| Fatty acid metabolism (4) | | | |  |
| *CUT1* | Unigene0004857 | Forward: CAGGCGTTCGAGCATTTCTG | 154 |  |
|  |  | Reverse: GCTCGTACCATAGCGAGGAC |  |  |
| *Acot9* | Unigene0028027 | Forward: AAGAAGCTGAAGCGAGGAGC | 167 |  |
|  |  | Reverse: TGTATCCCGCAGGAGAATGC |  |  |
| *AIM1* | Unigene0021159 | Forward: TAGCCATCGCCATTTTTGCG | 160 |  |
|  |  | Reverse: ATTGAGACATCCCCAGTCGC |  |  |
| *KCR1* | Unigene0018170 | Forward: TTCTTCCCGGGATGTTGACG | 164 |  |
|  |  | Reverse: GCCTTTGCCCTTGTACTCCA |  |  |
| Dark gland (8) | | | | |
| *PKSA* | Unigene0038693 | Forward: GGTTGAGATGGCTGTTGAAGC | 161 |  |
|  |  | Reverse: GACGGACATCACTCCTTAGGC |  |  |
| *PKSG5* | Unigene0036545 | Forward: ATTCGAGCTAGTGAAGGGCG | 132 |  |
|  |  | Reverse: AGGATGTTGTTGGCTAGGGC |  |  |
| *CHS* | Unigene0036546 | Forward: AAGTGGGACGACCTCAATGG | 144 |  |
|  |  | Reverse: CCACATGTTGCCGTAGTTGC |  |  |
| *CHS1* | Unigene0028641 | Forward: ACGACATAGACAGGCGCATC | 120 |  |
|  |  | Reverse: GGTGGACCACGTAGAAGAGC |  |  |
| *FGRAMPH1_01T20223* | Unigene0028167 | Forward: GGCTACTTGCTTTGTGTCGG | 167 |  |
|  |  | Reverse: GCAAAATGGGCAATGATGCG |  |  |
| *MALD1* | Unigene0006206 | Forward: TCCAAGGTCAAAGCCCACAG | 131 |  |
|  |  | Reverse: TTGATAGTTCCGACACCGCC |  |  |
| *STH-2* | Unigene0004263 | Forward: GGCACAAGGTCGTCCCTAAG | 109 |  |
|  |  | Reverse: AACCCGTCAACAAATTCGGC |  |  |
| *At4g20800* | Unigene0034254 | Forward: AACCAACGCGACAACTTCCT | 109 |  |
|  |  | Reverse: CGAAAACGGACTCGAAAGACG |  |  |

Note: The coding sequences of genes are shown in Table S3.

**Table S3** The coding sequences (CDS) of the selected 32 genes. (Note, the Table S3 was provided as a separated Excel document).

**Table S4** The RNA quality assessed using an Ultramicro spectrophotometer.

| Treatment (°C) | Concentration (μg/mL) | A260 | A280 | 260/280 |
| --- | --- | --- | --- | --- |
| 15 | 661.75±11.89 | 15.54±0.30 | 7.69±0.44 | 2.03±0.14 |
| 22 | 647.38±9.07 | 15.35±0.49 | 7.86±0.07 | 1.95±0.05 |

Values are average with their standard deviations (n = 3).
